# Nutritional and health factors influencing first antenatal visit among pregnant women in Indonesia

**DOI:** 10.3389/fpubh.2025.1715230

**Published:** 2025-12-04

**Authors:** Andjar Prasetyo, Iswari Hariastuti, Sri Sugiharti, Urip Tri Wijayanti, Hery Wahyudianto, Noviati Fuada, Dwi Hapsari Tjandrarini, Ina Kusrini, Yekti Widodo, Rika Rachmawati, Salimar, Budi Setyawati

**Affiliations:** 1 Regional Development Planning Agency of Magelang City, Magelang, Indonesia; 2 Center for Population Research, National Research and Innovation Agency, Jakarta, Indonesia; 3 Regional Development Planning Agency of Papua Province, Jayapura, Indonesia; 4 Organization for Health, National Research and Innovation Agency, Jakarta, Indonesia

**Keywords:** antenatal visit, chronic energy deficiency, malnutrition, weight gain, growth failure, pregnant women’s health, linear regression

## Abstract

This study evaluated the association between the health conditions of pregnant women, such as chronic energy deficiency, malnutrition, weight gain, and growth failure, with the frequency of first antenatal visits. A linear regression design was used with secondary data from 38 provinces in Indonesia in 2024. Predictor variables included chronic energy deficiency, malnutrition, weight gain, and growth failure. Data analysis using linear regression techniques showed a very strong regression model with an R value of 0.943 and an R^2^ of 0.889. An Adjusted R^2^ of 0.876 indicates an excellent model in explaining data variations. The variable of chronic energy deficiency had a coefficient of −0.769, malnutrition had a coefficient of 0.574, weight gain had a coefficient of −0.264, and growth failure had a coefficient of 0.307. These findings emphasize the importance of improving the nutrition and health condition of pregnant women to increase the frequency of first antenatal visits. Proper nutrition intervention programs can help prevent malnutrition and chronic energy deficiency, positively impacting the frequency of first antenatal visits. This study uniquely highlights the relationship between the health condition of pregnant women and the frequency of first antenatal visits in Indonesia.

## 1 Introduction

Timely and comprehensive first antenatal care (ANC K1) is a critical entry point for preventing maternal and child health complications, including stunting, anemia, low birth weight, and gestational disorders (1–3). ANC K1 enables early risk identification, nutritional support, immunization, and health education—key interventions aligned with Sustainable Development Goal (SDG) 3 (good health and well-being). Given the strong links between maternal health, socioeconomic equity, and intergenerational well-being, improving ANC K1 utilization is not only a clinical priority but also a matter of economic and social justice, directly supporting SDG 10 (Reduced Inequalities) and SDG 5 (Gender Equality). Despite high national ANC coverage in Indonesia (96.9% in 2023), many pregnant women do not receive optimal or timely ANC K1, particularly during the first trimester (4). Suboptimal use of ANC services—especially among women with chronic energy deficiency, malnutrition, inadequate weight gain, or growth failure—increases risks of adverse pregnancy outcomes (5, 6). In Ethiopia, only 20% of women initiate ANC in the first trimester, reflecting persistent barriers linked to education, wealth, and regional disparities (3, 7). These challenges underscore systemic inequities in access to and quality of maternal care, disproportionately affecting marginalized populations and undermining progress toward sustainable health equity.

While existing studies recognize the importance of ANC K1 and identify sociodemographic determinants (e.g., education, wealth, counseling) (8–10), few have specifically examined how maternal health conditions—such as chronic energy deficiency, malnutrition, and inadequate gestational weight gain—influence the timing and quality of ANC K1 in Indonesia. Moreover, the intersection of these health indicators with structural inequalities (e.g., rural–urban divides, gender-based disparities, and intergenerational health gaps) remains underexplored within a sustainable development framework. Current literature lacks an integrated, interdisciplinary perspective that connects individual-level health status with broader economic and systemic inequities as envisioned by the SDGs. This study addresses a critical gap by investigating the relationship between maternal nutritional and health status and ANC K1 utilization in Indonesia through the lens of sustainable development. By linking individual health conditions to systemic inequities, the findings will inform targeted, equity-focused policies that promote early and effective antenatal care. The research contributes to SDG 3 by enhancing maternal and child health outcomes, supports SDG 10 by highlighting disparities in healthcare access, and advances SDG 17 through evidence for integrated, multisectoral strategies. Ultimately, it provides actionable insights for strengthening sustainable, inclusive health systems that prioritize the most vulnerable—aligning health equity with economic justice and long-term resilience.

## 2 Method

This study employs a quantitative, ecological design using multiple linear regression (11) to examine the association between ANC K1—the percentage of pregnant women in a province receiving their first antenatal care visit—and four predictor variables: chronic energy deficiency (MUAC <23.5 cm), undernutrition (BMI < 18.5 kg/m^2^), adequate gestational weight gain (per Indonesia’s Ministry of Health trimester-specific guidelines), and intergenerational nutritional stress (proxied by provincial stunting prevalence in children under five, height-for-age <−2 SD). All variables are derived from the 2023 Indonesian National Health Survey (12), ensuring standardized, province-level measurement across Indonesia’s 38 provinces with complete data. Purposive sampling was used to include only provinces with full data availability, capturing diverse maternal health and nutritional profiles. Multiple linear regression is appropriate for this aggregated, proportion-based data, as it estimates the independent effect of each predictor on ANC K1 while accounting for intercorrelations, thereby informing evidence-based, equity-oriented public health policies. The regression equation obtained is as follows:


$$\begin{array}{l} \mathrm{ANC}\;\mathrm{K1}=\beta_{0}+\beta_{1}\times \text{Weight gain}+\beta_{2}\times \text{Fails to grow}\\ +\beta_{3}\times \text{Undernutrition}+\beta_{4}\times \text{Chronic lack of energy} \end{array}$$ (1)

Where β_0_ is a constant (intercept); β_1_ is the coefficient for the weight gain variable; β_2_ is the coefficient for the fails to grow variable; β_3_ is the coefficient for the undernutrition variable; and β_4_ is the coefficient for the chronic lack of energy variable. This technique is used to test the relationship between dependent variables and predictor variables. The analysis procedure includes: (1) Calculating the correlation coefficient (R) and determination coefficient (R^2^) to evaluate the strength and fit of the regression model; (2) Using the ANOVA test to determine the significance of the regression model as a whole; (3) Calculate the regression coefficient for each predictor variable and assess its significance through the t-test (13).

All statistical analyses were conducted using JASP version 0.18.3 (JASP Team, University of Amsterdam). The threshold for statistical significance was set at *p* < 0.05. Diagnostic tests were performed to assess regression assumptions. Multicollinearity was examined using the Variance Inflation Factor (VIF) and tolerance values generated in JASP, with all VIFs below 10 and tolerances above 0.1, indicating acceptable levels. The normality of residuals was evaluated using the Shapiro–Wilk test and Q–Q plots, while homoscedasticity was verified using the Breusch–Pagan test. Although the model yielded a high coefficient of determination (R^2^ = 0.889), further validation was carried out using the condition index to confirm that no severe multicollinearity was present (condition index < 30). Given the ecological nature of the dataset, causal inference cannot be established; the results should be interpreted as associations at the provincial level. Potential unmeasured confounders, such as socioeconomic status, healthcare accessibility, and regional disparities in service delivery, may influence ANC K1 coverage and are acknowledged as study limitations.

## 3 Result

Supplementary data reveal stark provincial disparities in maternal health indicators across Indonesia. ANC K1 coverage is highest in Bali (99.6%), Jakarta (99.5%), and Yogyakarta (99.2%), but lowest in Papua Mountains (58.7%) and Central Papua (63.6%). The Papua region also bears the highest burdens of chronic energy deficiency (Papua Mountains: 84.6%; Central Papua: 82.1%), undernutrition (Central Papua: 80.3%; Papua Mountains: 77.1%), and child stunting (Central Papua: 69.3%; Papua Mountains: 51.4%). In contrast, Java provinces—particularly Yogyakarta, Central Java, and West Java—consistently show better outcomes across all indicators. Notably, despite high energy deficiency and malnutrition, Papua mountains reports relatively high gestational weight gain (59.6%), suggesting complex nutritional dynamics. These findings highlight an urgent need for targeted maternal and child health interventions in the Papua region to address deep-seated inequities and improve outcomes (see Table 1).

**Table 1:** Distribution of variables by province in Indonesia (%).

Province	ANC K1	Chronic lack of energy	Undernutrition	Weight gain	Fails to grow
Aceh	95.6	68.8	66	51.3	16.4
North Sumatra	92.8	63.6	58.3	59.7	16.9
West Sumatra	97.7	59.8	59	57.3	18.2
Riau	95	62.3	61.1	51.9	16
Jambi	95.9	74.7	69.9	56.6	22.3
South Sumatra	96.5	62.1	56.6	54	14.5
Bengkulu	97.2	65.6	69.9	55.3	19.6
Lampung	97.6	60	59.3	55.1	13.1
Bangka Belitung Islands	97.4	64.2	67.9	59.7	18.9
Riau Islands	97.3	58.9	55.9	56.8	9.9
Jakarta	99.5	49.6	49.8	54.2	9.4
West Java	98.4	41.7	52	46.4	9.7
Central Java	98.9	48.2	49.1	50.4	7.3
In Yogyakarta	99.2	42.6	47.5	49.6	7.2
East Java	98.1	46.6	45.6	53.8	10.9
Banten	97	51.1	55.3	51.3	10.9
Bali	99.6	59	61	54.5	19.4
West Nusa Tenggara	99	50.1	53.9	44.6	11
East Nusa Tenggara	96.2	57.2	50.7	49.5	15.6
West Kalimantan	94.9	61.9	60.4	55.6	16.6
Central Kalimantan	92.2	69.9	65.1	52	20.2
South Kalimantan	97.4	55.7	53.3	50.8	13.5
East Kalimantan	98.4	55.5	59.4	59.7	18.6
North Kalimantan	98.8	72.2	69.5	60.4	21
North Sulawesi	96.8	63.8	62.9	63.9	18.9
Central Sulawesi	95.5	46.8	47.8	45.5	10.2
South Sulawesi	96.9	52.6	57.2	49.6	14.2
Southeast Sulawesi	94.2	65.4	63.7	52.7	17.3
Gorontalo	96.5	50.7	56	50.6	16.9
West Sulawesi	95.3	54.9	57.7	51.2	14.5
Maluku	87.2	65.2	59.8	46.9	16.1
North Maluku	90.6	71.6	66.5	56.6	26.2
West Papua	86.7	72.4	70.9	64.4	36
Southwest Papua	86	63	54.1	44.3	16.7
Papua	87	69.5	72.9	63.5	34.3
South Papua	81.1	60.1	70.8	44	26.7
Central Papua	63.6	82.1	80.3	59.6	69.3
Papua Mountains	58.7	84.6	77.1	54.6	51.4

The model summarized in Table 2, for the correlation coefficient (R) of 0.943 shows a very strong relationship between the dependent variable (ANC K1) and the predictors (chronic lack of energy, Undernutrition, weight gain, fails to grow). An R^2^ value of 0.889 indicates that 88.9% of the variation in the dependent variable (ANC K1) can be explained by the predictor variable in the model. It shows an excellent model in explaining data variations.

**Table 2:** Model summary, ANOVA^a^, and coefficients^a^.

Model summary				
Model	R	R^2^	Adjusted R^2^	Std. error of the estimate
1	0.943^a^	0.889	0.876	3.11241

ANOVA^a^						
Model		Sum of squares	df	Mean square	F	Sig.
1	Regression	2570.591	4	642.648	66.341	0.000^b^
	Residual	319.674	33	9.687		
	Total	2890.266	37			

Coefficients^a^								
Model		Unstandardized coefficients		Standardized coefficients	t	Sig.	Collinearity statistics	
		B	Std. error	Beta			Tolerance	VIF
1	(constant)	74.661	6.455		11.567	0.000		
	Weight gain	−0.264	0.116	−0.302	−2.280	0.029	0.191	5.230
	Fails to grow	0.307	0.150	0.295	2.047	0.049	0.161	6.207
	Undernutrition	0.574	0.111	0.352	5.169	0.000	0.721	1.386
	Chronic lack of energy	−0.769	0.076	−1.034	−10.070	0.000	0.318	3.145

a Predictors: (constant), chronic lack of energy, undernutrition, weight gain, fails to grow.

b Dependent variable: ANC K1.

Source: results analysis, 2025.

An Adjusted R^2^ value of 0.876 corrects the R^2^ for the number of predictors in the model (Equation 1), providing a more accurate measure of how well the general model can be applied to a wider population. A high Adjusted R^2^ (87.6%) still indicates that the model is very robust. The estimated standard error value of 3.11241 indicates the standard error rate in predicting the ANC K1 value. The smaller this value, the better the model will be at predicting the dependent variables. This regression model is very robust and can explain most of the variations in ANC K1 based on the predictor variables (chronic lack of energy, Undernutrition, weight gain, fails to grow). High R, R^2^, and Adjusted R^2^ values indicate that the model has a good match with the data, while a low standard error value indicates good prediction accuracy.

The ANOVA results (Table 2) indicate that the regression model significantly explains variation in ANC K1. The total sum of squares (2,890.266) reflects overall variability in ANC K1 across provinces. Of this, the model accounts for 2,570.591, while 319.674 remains unexplained (residual). The high *F*-value (66.341) and a *p*-value of 0.000 confirm the model’s overall significance (*p* < 0.05), meaning at least one predictor—chronic energy deficiency, undernutrition, weight gain, or stunting (“fails to grow”)—is significantly associated with ANC K1. The final regression equation is:


$$\begin{array}{l} \mathrm{ANC}\;\mathrm{K1}=97.397–0.769\;(\text{chronic energy deficiency})\\ -0.738\;(\text{undernutrition})-0.625\;(\text{weight gain})-2.526\;(\text{fails to grow}) \end{array}$$

All coefficients are unstandardized (B), showing the expected change in ANC K1 for a one-percentage-point increase in each predictor, holding others constant. The intercept (97.397) represents the estimated ANC K1 when all predictors are zero. Standardized beta coefficients (not shown here but referenced) would indicate the relative strength of each predictor’s influence, adjusted for scale differences. Together, these results demonstrate that maternal and child nutritional indicators significantly and negatively influence timely ANC uptake, with stunting showing the strongest adverse association.

The regression analysis reveals mixed associations between maternal and child nutritional indicators and ANC K1 utilization. Chronic energy deficiency is strongly and negatively associated with ANC K1 (*β* = −1.034, *p* < 0.001), indicating that provinces with higher rates of energy deficiency have significantly lower ANC K1 coverage. In contrast, undernutrition (β = 0.352, p < 0.001) and child stunting (“fails to grow,” β = 0.295, *p* = 0.049) show positive associations with ANC K1, suggesting that pregnant women in areas with higher malnutrition or intergenerational growth failure may be more likely to initiate antenatal care—possibly due to heightened health awareness or targeted interventions. Surprisingly, adequate gestational weight gain is negatively linked to ANC K1 (*β* = −0.302, *p* = 0.029), implying that better nutritional outcomes may coincide with delayed care initiation. Multicollinearity is moderate to high for some variables (VIF up to 6.21), but all predictors remain statistically significant. Overall, the findings suggest that while severe nutritional deprivation—particularly chronic energy deficiency—hinders early ANC access, other forms of malnutrition may trigger earlier care-seeking behavior, highlighting the complex relationship between nutritional status and antenatal service utilization in Indonesia.

## 4 Discussion

This study found that adequate gestational weight gain was negatively associated with ANC K1, while maternal undernutrition and child stunting (“growth failure”) were positively linked to earlier ANC initiation—suggesting that perceived or actual nutritional risk may prompt earlier care-seeking. This aligns with Indonesian studies showing that inadequate weight gain, pregnancy complications, and LBW risk drive ANC use (14–18), and that community programs can accelerate first visits (19). In contrast, chronic energy deficiency strongly reduced ANC K1, likely reflecting systemic access barriers in the most vulnerable regions. These patterns are consistent with global evidence linking malnutrition, poverty, and healthcare inequity to antenatal care utilization (20–22), as well as clinical literature emphasizing nutrition’s role in maternal and child health outcomes (23–25).

The study found that pregnant women with children experiencing growth failure, maternal malnutrition, or chronic energy deficiency tended to initiate ANC K1 more frequently, likely due to heightened awareness of risk and the need for closer medical monitoring. Fetal growth restriction is a known predictor of adverse outcomes, including perinatal mortality, underscoring the importance of early detection and intervention (26–28). Conditions like hypertension, congenital heart disease, and placental abnormalities further elevate risks, necessitating intensified antenatal care (29–31), while gaps in care—such as in India, where many high-risk women lacked adequate ANC—highlight systemic shortcomings (32). Similarly, maternal undernutrition increases susceptibility to complications, driving earlier care-seeking; this is prevalent across diverse settings, from Sub-Saharan Africa and Ethiopia to Bangladesh and Sierra Leone, where food insecurity, poverty, anemia, and malaria exacerbate nutritional deficits (33–38). Chronic energy deficiency (CED), often linked to low BMI or MUAC, is a major risk factor for low birth weight and stunting in Indonesia, influenced by diet, income, education, and pandemic-related disruptions (39–44). Together, these findings indicate that poorer maternal and child nutritional status—manifested as CED, undernutrition, or stunting—acts as both a clinical trigger and a structural signal for earlier ANC initiation, reinforcing the need for targeted, responsive antenatal programs that address underlying inequities and provide timely support to high-risk pregnancies. These findings are important to know the potential causes and important steps to take, which are summarized in Table 3.

**Table 3:** Statistical findings, causes and important steps from the results of the study.

No.	Variable	Findings.	Cause	Important steps
1.	Weight gain	Pregnant women who experience less weight gain tend to have ANC K1 more often.	Inadequate weight gain may signal health problems or nutritional deficiencies that make pregnant women more aware of the need for health monitoring to prevent complications.	Increasing education about the importance of healthy weight gain during pregnancy and providing adequate nutritional support.
2.	Fails to grow	Pregnant women with children who have growth failure are more likely to do ANC K1.	Growth failure may indicate a developmental or health problem in the fetus, prompting the mother to seek medical monitoring to identify and treat the problem early.	Strengthen monitoring and early intervention programs for fetal growth problems and provide more intensive medical support for mothers experiencing these problems.
3.	Undernutrition	Pregnant women who are malnourished tend to do ANC K1 more often.	Malnutrition can lead to concerns about maternal and fetal health, which increases the frequency of visits to health facilities to address nutritional issues.	Provide better nutrition and supplementation programs for pregnant women and increase access and education about a balanced diet.
4.	Chronic lack of energy	Pregnant women who experience chronic energy deficiency tend to do ANC K1 more often.	Chronic energy deficiency may indicate the presence of a more serious health problem that requires more frequent medical attention to prevent negative impacts on pregnancy.	Identify and address the causes of energy deprivation, including underlying health issues, as well as increase support for the mental and physical health of pregnant women.

Source: author’s analysis, 2025.

This study advances health behavior theory by demonstrating how maternal health conditions—particularly inadequate weight gain, malnutrition, and chronic energy deficiency—influence antenatal care (ANC) utilization, aligning with the health trust model. The findings underscore the need for healthcare providers to tailor ANC for high-risk pregnant women and for policymakers to design targeted interventions ensuring regular ANC attendance. Public health efforts should also promote adequate nutrition and energy intake during pregnancy to enhance maternal and fetal outcomes. Future research should include longitudinal and intervention studies to assess how changes in nutritional and energy status affect ANC use and health outcomes, explore underlying mechanisms (including psychosocial and access-related factors), and validate findings across diverse, culturally varied populations to inform equitable, evidence-based maternal health strategies.

This study provides a novel contribution by integrating maternal and child nutritional indicators into a single ecological model predicting first antenatal care visits (ANC K1) across Indonesia’s 38 provinces. Unlike previous studies that examined individual-level or localized determinants, this research offers a province-level, cross-sectional perspective linking intergenerational nutrition to ANC initiation. This approach advances current knowledge by demonstrating how aggregate nutritional status functions as a contextual determinant of health service utilization, bridging gaps between nutrition surveillance and maternal health policy. Moreover, it aligns with recent global analyses emphasizing integrated maternal–child nutrition approaches to improve early care utilization (45).

The findings can be conceptually interpreted using Andersen’s Behavioral Model of Health Services Use, where predisposing (nutritional awareness), enabling (access to services), and need factors (perceived health risk) jointly shape ANC-seeking behavior. Similarly, the Health Belief Model supports the interpretation that higher perceived susceptibility and severity of nutritional risk motivate earlier ANC initiation among undernourished or high-risk women. Conversely, limited enabling factors such as poverty or geographic barriers may explain lower ANC initiation among women with chronic energy deficiency. These theoretical interpretations are consistent with recent public health evidence highlighting behavioral and contextual determinants of healthcare access in low-resource settings (46) and community-level enablers of maternal care utilization (47).

This study is limited by its ecological design, which precludes causal inference and may be influenced by unmeasured confounders such as household income, education, and access to health services. Nonetheless, the results provide important policy insights: strengthening maternal nutrition programs, improving ANC outreach in nutritionally vulnerable provinces, and integrating nutrition-sensitive indicators into routine ANC monitoring could enhance maternal and fetal health outcomes. Future research should employ multilevel or longitudinal designs to further disentangle causal pathways and evaluate policy interventions that address both behavioral and structural barriers to ANC utilization.

## 5 Conclusion

The study investigated how various health factors of pregnant women, such as weight gain, growth failure, malnutrition, and chronic energy deficiencies, affect the frequency of first ANC K1. The results showed that weight gain had a negative association with ANC K1 visits (coefficient = −0.625, standard error = 0.294, t = −2.124, *p* = 0.041), where adequate weight gain reduced the frequency of visits. In contrast, growth failure was positively associated with ANC K1 (coefficient = 0.307, standard error = 0.150, t = 2.047, *p* = 0.049), suggesting that fetal growth problems drove more visits. Malnutrition was also positively associated with ANC K1 (coefficient = 0.574, standard error = 0.111, t = 5.169, *p* = 0.000), reflecting the need for more intensive monitoring for mothers who are malnourished. Chronic energy deprivation had a very strong negative association with ANC K1 (coefficient = −0.769, standard error = 0.076, t = −10.070, *p* = 0.000), indicating that severe energy deprivation requires more health visits. Statistical results support this conclusion with an R^2^ of 0.889 and an Adjusted R^2^ of 0.876, which suggests that 88.9% of the variation in the frequency of ANC K1 can be explained by this model. An *F*-value of 66,341 (*p* = 0,000) also indicates that the model is statistically significant. These findings confirm the purpose of the study to understand the impact of maternal health conditions on antenatal visits. These findings are also in line with previous research and expand the understanding of how health conditions affect pregnant women’s health-seeking behavior.

## References

[1] Gunawan P Basyir V Serudji J. Factors associated with first antenatal care visit (K1) in pregnant women at the Mungka health center, Lima Puluh Kota. Int J Res Rev. (2024) 10:824–33. doi: 10.52403/ijrr.20231282

[2] Herlinda H Elvina A Rismayani R Linshia L. Analysis of the regularity of antenatal care (ANC) visits with anemia in pregnant women. JKM (Jurnal Kebidanan Malahayati). (2024) 10:552–8. doi: 10.33024/jkm.v10i6.15021

[3] Kidie AA Asmamaw DB Belachew TB Fetene SM Baykeda TA Endawkie A . Socioeconomic inequality in timing of ANC visit among pregnant women in Ethiopia, 2019. Front Public Health. (2024) 12. doi: 10.3389/fpubh.2024.1243433, PMID: 38550321 PMC10972848

[4] Ariningtyas RE Yuspita Sari R Sevtiyani I Raharjo UD. Evaluation of the completeness antenatal care coverage (K1-K4) in the special region of Yogyakarta Province. PROMOTOR. (2024) 7:403–7. doi: 10.32832/pro.v7i3.699

[5] Indriyani D Yunitasari E Efendi F. Factors associated with adequate antenatal care among pregnant women in rural Indonesia. Afr J Nurs Midwifery. (2024). doi: 10.25159/2520-5293/14232

[6] Sari DP Ekoriano M Pujihasvuty R Kistiana S Nasution SL Ardiana I . Antenatal care utilization on low birth weight children among women with high-risk births. F1000Res. (2024) 12:399. doi: 10.12688/f1000research.126814.2, PMID: 38434626 PMC10905146

[7] Shiferaw D Feyisa BR Biru B Yesse M. Antenatal care component utilization and associated factors among pregnant women in Ethiopia: multilevel analysis of Ethiopian Mini demographic and health survey 2019. PLoS One. (2024) 19:e0303118. doi: 10.1371/journal.pone.0303118, PMID: 38809897 PMC11135780

[8] Yuniwati C Fithriany CN Kartika L Harahap S Dewi S. Analysis of factors affecting pregnancy visits for mother and fetal health. Health Technol J. (2023) 1. doi: 10.53713/htechj.v1i3.48

[9] Feyisa GC Dagne A Woyessa D Ephrem T Ahmed A G/Senbet H . Mean difference in timing of first antenatal checks across regions and associated factors among pregnant women attending health facilities in Ethiopia: evidence from Ethiopian demographic health survey, 2019. BMC Public Health. (2023) 23:2393. doi: 10.1186/s12889-023-17356-2, PMID: 38041108 PMC10693148

[10] Suebu LJ Usman AN Ahmad M. Achievement of antenatal care visit K4 pregnant women with the support of health workers: a literature review. Nurse Health. (2022) 11:172–81. doi: 10.36720/nhjk.v11i1.349

[11] Edward Elgar Publishing. Regression analysis In: Handbook of research methods in international relations. Cheltenham, UK: Edward Elgar Publishing (2022). 447–67.

[12] Pusat Kebijakan Upaya Kesehatan (2024). Survey Kesehatan Indonesia 2023 Dalam Angka Badan Kebijakan Pembangunan Kesehatan, Kementerian Kesehatan Republik Indonesia. Available online at: https://www.badankebijakan.kemkes.go.id/hasil-ski-2023/

[13] Eye A Wiedermann W. Correlation and regression analysis In: The general linear model. Cambridge, UK: Cambridge University Press (2023). 10–123.

[14] Naim S Sulistyorini Y Evriyanto Y Yuniati E. The correlation between low birthweight (LBW) with infant mortality rates (IMR) and antenatal care (ANC) in East Java 2018. Jurnal Biometrika Dan Kependudukan. (2020) 9:121. doi: 10.20473/jbk.v9i2.2020.121-129

[15] Adawiyah AR Djokosujono K Alam N Setiawati NA. An effective method to predict low birth weight in Indonesia rural area. Indones J Public Health Nutr. (2021) 2. doi: 10.7454/ijphn.v2i1.5307

[16] Maulidani M Abdullah A Nurjannah N. Analisis Hubungan Komplikasi Kehamilan Dengan Kejadian Bayi Berat Lahir Rendah di Indonesia (Analisis Data Sekunder Survei Demografi Kesehatan Indonesia Tahun 2017). Jurnal Kesehatan Komunitas. (2023) 9:332–42. doi: 10.25311/keskom.vol9.iss2.1359

[17] Meiriza W Aladin A Edison E. The correlation of maternal factors and the quality of antenatal care services with low birth weight babies in health facilities level I. J Midwifery. (2018) 3:103. doi: 10.25077/jom.1.1.103-114.2018

[18] Yuwana NRDA Mahmudiono T Rifqi MA. Faktor-Faktor yang Berhubungan dengan Kejadian Bayi Berat Lahir Rendah (BBLR) di Indonesia Berdasarkan Analisa Data Sekunder SDKI Tahun 2017. Media Gizi Kesmas. (2022) 11:451–7. doi: 10.20473/mgk.v11i2.2022.451-457

[19] Paratmanitya Y Helmyati S Nurdiati DS Lewis EC Gittelsohn J Hadi H. The effect of a maternal mentoring program on the timing of first antenatal care visit among pregnant women in Bantul, Indonesia: results of a cluster randomized trial. Health Promotion Perspect. (2021) 11:307–15. doi: 10.34172/hpp.2021.39, PMID: 34660225 PMC8501487

[20] Mahumud RA Sarker AR Sultana M Islam MN Hossain MR Hossain MG In: De UK Pal M Bharati P, editors. Issues on health and healthcare in India. Singapore: Springer (2018)

[21] Masoud S Menon P Bhutta ZA In: Preedy VR Patel VB, editors. Handbook of famine, starvation, and nutrient deprivation. Cham, Swiss: Springer International Publishing (2019).

[22] Siddiqui FJ Belayneh G Bhutta ZA In: Humphries DL Scott ME Vermund SH, editors. Nutrition and infectious diseases. Cham, Swiss: Springer International Publishing (2021).

[23] Buonocore G Bracci R Weindling M. Neonatology. Cham, Swiss: Springer International Publishing (2018).

[24] Goodyer P Phadke K In: Phadke KD Goodyer P Bitzan M, editors. Manual of pediatric nephrology. Berlin Heidelberg: Springer (2014)

[25] Rajagopal S Ramachandran S Sundararaman G Gadde Venkata S. Role of nutrients in neurological disorders. Singapore: Springer (2022).

[26] Unterscheider J O’Donoghue K Daly S Geary MP Kennelly MM McAuliffe FM . Fetal growth restriction and the risk of perinatal mortality-case studies from the multicentre PORTO study. BMC Pregnancy Childbirth. (2014) 14. doi: 10.1186/1471-2393-14-63, PMID: 24517273 PMC3923738

[27] Pattinson RC Hlongwane TMAG Vannevel V. Challenges to improve antenatal and intrapartum care in South Africa. S Afr Med J. (2019) 109:15–9. doi: 10.7196/SAMJ.2019.v109i11b.14248, PMID: 32252862

[28] El-Raggal NM Nawara MM Abd El-Ghani AM Zareif ME Shehata BM. Cord blood ischemia-modified albumin levels in preterm infants with intrauterine growth restriction. QJM Int J Med. (2021) 114. doi: 10.1093/qjmed/hcab113.038

[29] Keepanasseril A Pillai AA Yavanasuriya J Raj A Satheesh S Kundra P. Outcome of pregnancies in women with pulmonary hypertension: a single-Centre experience from South India. BJOG Int J Obstetr Gynaecol. (2019) 126:43–9. doi: 10.1111/1471-0528.15681, PMID: 30868706

[30] Dhiman S Sharma A Gupta A Vatsa R Bharti J Kulshrestha V . Fetomaternal outcomes in pregnant women with congenital heart disease: a comparative analysis from an apex institute. Obstet Gynecol Sci. (2024) 67:218–26. doi: 10.5468/ogs.23264, PMID: 38356351 PMC10948205

[31] Babic I Tulbah M Kurdi W. Antenatal embolization of a large placental chorioangioma: a case report. J Med Case Rep. (2012) 6. doi: 10.1186/1752-1947-6-183, PMID: 22759589 PMC3419096

[32] Dattaray C Mandal D Shankar U Bhattacharya P Mandal S. Obstetric patients requiring high-dependency unit admission in a tertiary referral Centre. Int J Crit Illn Inj Sci. (2013) 3:31–5. doi: 10.4103/2229-5151.109416, PMID: 23724382 PMC3665116

[33] Malel ZJ Jeremiah MM Augustine AA Machok ND Tong DT Madut MM . Pattern of malnutrition among pregnant women attending antenatal care service at juba teaching hospital, South Sudan. Adv Public Health. (2024) 2024. doi: 10.1155/2024/1877568

[34] Getaneh T Negesse A Dessie G Desta M Assemie MA Tigabu A. Predictors of malnutrition among pregnant women in Ethiopia: a systematic review and meta-analysis. Hum Nutr Metabol. (2021) 26. doi: 10.1016/j.hnm.2021.200131 PMC8654570 34901276

[35] Amin MR Azim A Haque M Ali M. Prevalence of child underweight, maternal malnutrition and pregnancy weight gain of urban poor of Dhaka and Chittagong, Bangladesh: an analysis of growth monitoring and promotion cards. Bangladesh J Nutr. (2018). doi: 10.3329/bjnut.v28i1.69884

[36] Bussink-Voorend D Bussink AP Falama AM Stekelenburg J. Health indicators of pregnant women in tonkolili district, rural Sierra Leone. Int J Environ Res Public Health. (2020) 17. doi: 10.3390/ijerph17113918, PMID: 32492879 PMC7312252

[37] Fakier A Petro G Fawcus S. Mid-upper arm circumference: a surrogate for body mass index in pregnant women. S Afr Med J. (2017) 107:606–10. doi: 10.7196/SAMJ.2017.v107i7.12255, PMID: 29025451

[38] Stefanova P Georgiev A Vasileva R Kostadinov S. Irrational nutrition as a risk factor during pregnancy and childbirth. Eur J Pub Health. (2023) 33. doi: 10.1093/eurpub/ckad160.1526

[39] Ginting KB Putra GL. Logistic regression approach to study pregnant women cases as a risk factor of LBW at the Batakte public health center, Kupang Barat District. BAREKENG. (2023) 17:1825–34. doi: 10.30598/barekengvol17iss4pp1825-1834

[40] Hariati MC Harefa K. The effect of mentoring by involving PKK mothers on weight control of pregnant women. Jurnal Kebidanan Kestra (JKK). (2023) 5:229–35. doi: 10.35451/jkk.v5i2.1654

[41] Yuliarti Y Ermi N Arinda DF. The causes of malnutrition for pregnant mothers analysis and the impact on the COVID-19 pandemic. Jurnal Ilmu Kesehat Masy. (2022) 13:210–23. doi: 10.26553/jikm.2022.13.2.210-223

[42] Marjuang Purba E Baning Rahayujati T Hakimi M. Kehamilan usia remaja dan kejadian bayi berat lahir rendah di Kabupaten Gunungkidul. BKM J Commun Med Public Health. (2016) 32. doi: 10.22146/bkm.7645

[43] Fathurahman M. Modeling count data with over-dispersion using generalized Poisson regression: a case study of low birth weight in Indonesia. Jurnal Statistika Universitas Muhammadiyah Semarang. (2023) 11:45. doi: 10.26714/jsunimus.11.1.2023.45-60

[44] Sandra C. Penyebab kejadian kekurangan energi kronis pada ibu hamil risiko tinggi dan pemanfaatan antenatal care di wilayah kerja puskesmas jelbuk jember. J Adm Kesehat Indones. (2018) 6:136. doi: 10.20473/jaki.v6i2.2018.136-142

[45] Ding L Zhang M Strodl E Yin X Wen G Sun D . The effects of early childhood probiotic intake on the association between prenatal micronutrient supplementation and neurobehavioral development in preschool children: a four-way decomposition analysis. Front Nutr. (2025) 12. doi: 10.3389/fnut.2025.1614820, PMID: 40469665 PMC12133521

[46] Ahun MN Aboud F Wamboldt C Yousafzai AK. Implementation of UNICEF and WHO’S care for child development package: lessons from a global review and key informant interviews. Front Public Health. (2023) 11. doi: 10.3389/fpubh.2023.1140843, PMID: 36875409 PMC9978394

[47] Bhuiyan MI Haque MA. NGOs’ initiatives and grassroots approach for accessing to health care services for the slum people in Dhaka. Front Health Serv. (2024) 4:1386698. doi: 10.3389/frhs.2024.1386698, PMID: 39364142 PMC11446876

